# The application of alkaline and acidic electrolyzed water in the sterilization of chicken breasts and beef liver

**DOI:** 10.1002/fsn3.305

**Published:** 2015-11-01

**Authors:** Yuko Shimamura, Momoka Shinke, Miki Hiraishi, Yusuke Tsuchiya, Shuichi Masuda

**Affiliations:** ^1^ School of Food and Nutritional SciencesUniversity of Shizuoka52‐1 YadaSuruga‐kuShizuoka422‐8526Japan

**Keywords:** Acidic electrolyzed water, alkaline electrolyzed water, beef liver, chicken meat

## Abstract

The sterilization effect of a combination treatment with alkaline electrolyzed water (AlEW) and strong acidic electrolyzed water (StAEW) on fresh chicken breasts and beef liver was evaluated. Samples (1, 5, and 10 g) were inoculated with *Salmonella* Enteritidis NBRC3313, *Escherichia coli* ATCC 10798, *Staphylococcus aureus* FDA209P, and *S. aureus* C‐29 [staphylococcal enterotoxin A (SEA) productive strain] and subjected to a dipping combination treatment (4°C and 25°C for 3 min) with AlEW and StAEW. Combination treatment with AlEW and StAEW significantly reduced the bacteria, and reduction of more than 1 log colony‐forming units (CFU)/g was achieved. Furthermore, this combination treatment significantly decreased the SEA gene expression level in samples. Some quality variables of the meat samples such as pH, lipid oxidation, color, amino‐acid content, texture, and sensory characteristics showed no significant differences between the combination treatment with AlEW and StAEW and the untreated control.

## Introduction

Among the poultry meat, chicken is a very popular food source around the world (Chouliara et al. 2007). Because chicken meat is highly perishable, it is important to ensure that chicken meat products are not microbial contaminated during storage and marketing (Hong et al. 2008a,b). It is reported that *Salmonella*,* Listeria*,* Campylobacter*,* Escherichia coli,* and *Staphylococcus aureus* in chicken meat cause food poisoning (Kitai et al. 2005; Hong et al. 2008a,b). Particularly, Japanese people have the custom of consuming raw chicken sashimi called ‘torisashi’. In Japan, raw beef liver, often served dipped in soy sauce or sesame oil, is a popular dish at Japanese beef barbecue restaurants. With such a background, a massive food‐poisoning outbreak caused by a raw beef dishes Yukhoe contaminated with enterohemorrhagic *Escherichia coli* O111:H8 and 72 O157:H7 occurred in Japan in 2011. This outbreak resulted in the 181 patients, including 21 patients with acute encephalopathy and the death of five patients with hemolytic‐uremic syndrome (Watahiki et al. 2014).

Japanese beef barbecue restaurants banned the serving of raw liver after the aforementioned food poisoning incident. In April of 2012, a food safety panel under the Cabinet Office supported the plan to restrict the serving of raw beef liver. Subsequently, in June of 2015, Ministry of Health, Labour and Welfare has banned serving raw pork, following a similar ban in 2012 on beef liver. Although new regulations pertaining to fresh meat were enforced by the Ministry of Health, Labour and Welfare, effective sterilization methods have not yet been established. In addition, many Japanese citizens continue to want to consume raw meat and beef liver. Therefore, a novel method for reducing the initial number of bacteria in raw meat and beef liver is required.

To improve the microbial safety of chicken, various techniques have been used for the reduction of bacterial contaminants (González‐Fandos and Dominguez 2007; Kim and Day 2007; Hyeon et al. 2013). Some sterilization applications using electrolyzed water were reported. Koseki and Isobe demonstrated that a combination treatment of alkaline electrolyzed water (AlEW) and mild heat process reduced the *Escherichia coli* O157:H7 and *Salmonella* populations (Koseki and Isobe 2007). Recently, combinations of AlEW and citric acid showed a strong antimicrobial effect on background flora and foodborne pathogens on freshly cut produce or cereal grains (Park et al. 2009; Rahman et al. 2010). However, these applications were not suitable for sterilization of raw meat and beef liver. The sterilization application of raw meat samples using acidic electrolyzed water has also been reported (Rahman et al. 2012). However, the bacteriocide properties of acidic electrolyzed water become less noticeable by contact with organic matters from meat. It was reported that AlEW followed by acidic electrolyzed water achieved 4‐ to 5‐log reductions in *L. monocytogenes* biofilms formation, even in the presence of organic matter (Ayebah et al. 2006).

Therefore, we evaluated that the application of AlEW and strong acidic electrolyzed water (StAEW) in the sterilization of chicken breasts and beef liver. There is also the advantage that waste water becomes neutral by mixture with AlEW and StAEW. The current study examined the effect of a combination treatment with AlEW and StAEW on microbial growth, bacterial toxin gene expression level, pH, lipid oxidation, color, amino‐acid content, and sensory characteristics of fresh chicken and beef meat during storage.

## Materials and Methods

### Preparation of bacterial inoculum

*Salmonella enterica* subsp. *enterica* serovar Enteritidis NBRC3313, *E. coli* ATCC 10798, *Staphylococcus aureus* FDA209P, and *S*. *aureus* C‐29 [staphylococcal enterotoxin A (SEA) productive strain)] cultures maintained in our laboratory were used (Tsutsuura et al. 2013). These strains were subcultured on brain heart infusion (BHI) agar (Oxoid Ltd., London, UK). Each strain was incubated at 37°C with shaking for 16–18 h in 3 mL of BHI broth. After incubation, 30 *μ*L of fermented broth was transferred to 3 mL of BHI broth and incubated at 37°C with shaking for 16–18 h. Bacterial cells (1 mL) were collected by centrifugation at 6000 × *g* for 5 min, washed twice with a 1 mL of sterile phosphate buffered saline (PBS) and then resuspended in 1 mL of the same solution to obtain a final cell concentration of 10^9^ CFU/mL.

### Sample preparation

Fresh, raw boneless chicken breasts, beef liver, and beef round were obtained from a meat‐packing factory in Shizuoka, Japan and stored in a refrigerator at 4°C prior to use for the experiment within 3 h. Each sample portion was subdivided (1, 5, and 10 g) and used for microbial and bacterial toxin gene expression level, pH, lipid oxidation, color, amino‐acid content, and sensory analyses. Three different sizes of samples were used to check the requisite amount of the electrolyzed water for sterilization. Both inoculated and noninoculated samples by each strain were stored at 4°C before various tests. For a quality evaluation of the meat, noninoculated samples were stored at 4°C after treated with two types of electrolyzed water (EW) solutions, as described later.

### Preparation of treatment solutions

Alkaline electrolyzed water (AlEW; pH 11.5) and strong acidic electrolyzed water (StAEW; pH 2.5) used in this study, were produced by electrolysis of a dilute NaCl solution using Win‐G electrolysis device (Gaea Co. Ltd., Tokyo, Japan). Oxidation reduction potential (ORP) and available chlorine concentration (ACC) of StAEW at 4°C were 975 ± 15 mV and 30 ± 2.0 mg/L respectively. At room temperature (25°C), ORP and ACC were 960 ± 5 mV and 14 ± 1.5 mg/L respectively.

### Inoculation of samples and treatment

The samples (1, 5, and 10 g) on the skin side were inoculated with each bacteria (*S. *Enteritidis, *E*. *coli,* and *S*. *aureus*) at a level of 10^6^–10^7^ log CFU/g, and left to air dry for 15 min. Using this treatment, approximately 5 log CFU/g of bacteria were obtained on samples. The inoculated samples were then packaged in a stomacher bag and stored at 4°C prior to quality analysis. Physicochemical and sensorial attributes of noninoculated samples were studied.

### Procedures for disinfection

To check the requisite amount of the electrolyzed water for sterilization, a constant amount of AlEW and StAEW was used for three different sizes (1, 5, and 10 g) of samples. Each inoculated sample (1, 5, and 10 g) was dipped by shaking (165 rpm) in 100 mL of AlEW solution (pH 11.5) at room temperature for 3 min and then dipped in 100 mL of StAEW (pH 2.5) at 7.6 ± 1.2°C (preserved in cold storage) or at 25.2 ± 0.1°C (preserved at room temperature). The neutralized sample was dipped by shaking (165 rpm) in 100 mL of StAEW solution (pH 2.5) at room temperature for 3 min. After the sample was dipped in 100 mL of AlEW (pH 11.5), the bacterial population was counted. Survival rate in the samples of each bacteria were counted as −log [(CFU after treatment) / (CFU before treatment)].

### Microbiological analyses

After AlEW and StAEW treatment (day 0), all samples were aseptically and immediately placed in a stomacher bag containing 90 mL of sterile PBS and homogenized for 2 min with iMIX (Interlab; Monti Rina, Roma). After homogenization, 0.1 mL aliquots of the samples were serially diluted in 0.9 mL of sterile PBS as needed, and 0.1 mL of the appropriate dilutions were spread‐plated onto each selective medium. Total viable counts were determined by plating appropriately diluted samples onto BHI agar. Xylose lysine deoxycholate (XLD; Merck, Darmstadt, Germany), XM‐G (Nissui pharmaceutical Co., Ltd., Tokyo, Japan) and Mannitol salt agar (Eiken Chemical, Tokyo, Japan) were used for *Salmonella* spp., *E*. *col*i, and *S*. *aureus* respectively. All inoculated agar plates were incubated at 37°C for 1–2 days, following which the CFU levels were enumerated. The samples (inoculated) treated with sterilized water were used as control throughout the experiment.

### RNA isolation and real‐time polymerase chain reaction (RT‐PCR)

Each 10 ± 0.2 g sample was experimentally inoculated with *S. aureus* C‐29 and subjected to a dipping combination treatment (4°C for 3 min) with AlEW and StAEW. Both EW treated samples were aseptically and immediately placed in a stomacher bag containing 90 mL of sterile PBS and homogenized for 5 min with iMIX (Interlab). After homogenization, 2 mL aliquots of the samples were collected by centrifugation for 3 min at 4°C at 10,000 × *g* and stored at −80°C to prevent RNA degradation. Total RNA was purified using a RiboPure‐Bacteria kit (Ambion, Austin, TX), according to the manufacturer's instructions. The RT‐PCR was performed with SYBR Premix Ex Taq (Takara, Shiga, Japan) and RT‐PCR system Thermal Cycler Dice^®^ Real Time System Single (Takara). The RT‐PCR reaction mixture composed of 0.2 *μ*mol/L of each primer, 1× SYBR^®^ Premix EX Taq^™^ (Perfect Real Time) premix reagent (Takara) and 50 ng cDNA at a final volume of 25 *μ*L. Each sample was normalized to 16S rRNA expression. The primers used for the detection of *sea* and 16S rRNA were as described by Klotz et al. (2003) and Chang et al. (2006) respectively (Klotz et al. 2003; Chang et al. 2006). The following primers were used: sea forward (F), 5′‐AAAATACAGTACCTTTGGAAACGGTT‐3′ and *sea* reverse (R), 5′‐TTTCCTGTAAATAACGTCTTGCTTGA‐3′; 16S rRNA F, 5′‐GCGAAGAACCTTACCAAATC‐3′ and 16S rRNA R, 5′‐CCAACATCTCACGACACG‐3′. The RT‐PCR data were analysed using the $2^{-\Delta \Delta C_{t}}$ method described in Applied Biosystems, User Bulletin no. 2.

### pH measurement

Each sample (5 g) was homogenized in 45 mL of distilled water. Sample solutions were centrifuged at 2000 × *g* for 15 min, and the pH was measured using a pH meter (pH meter F‐21; HORIBA Ltd., Kyoto, Japan). Evaluation of pH was examined at day 0 and up to the 7 days after being vacuum‐packaged in a stomacher bag and placed in refrigerated storage at 4°C.

### Thiobarbituric acid reactive substances (TBARS) analysis

The lipid oxidation value of each sample was determined by the TBARS method. Each sample (10 g) was homogenized in 20 mL of 10% trichloroacetic acid. Sample solutions were centrifuged at 2300 × *g* for 30 min at 4°C. After centrifugation, supernatant solutions (2 mL) were then transferred into a disposable test tube and 2 mL of 20 mmol/L 2‐thiobarbituric acid solution was added. The mixture was vortexed and boiled in a water bath for 20 min and cooled at room temperature for 10 min. The absorbance of the resulting supernatant fluid was measured at 531 nm. Lipid oxidation evaluation value of each sample was examined at day 0 and up to 7 days under 4°C storage condition.

### Color measurement

The color of meat was confirmed by visual observation. Each inoculated sample was dipped in each 100 mL of AlEW and StAEW solutions for 0, 3, and 5 min. The color difference of meat was measured using the colorimeter CR‐300 (Konica Minolta, Tokyo, Japan) and expressed as color L*(brightness/darkness), a*(redness/greenness) and b* (yellowness/blueness) values. The colorimeter was calibrated throughout the study using a standard white ceramic tile. Color evaluation was examined at day 0 and up to the 7 days after being vacuum‐packaging in a stomacher bag and placed in refrigerated storage at 4°C.

### Free amino acid and dipeptide analysis

Each Sample (10 g) was homogenized in 40 mL of 2% (w/v) sulfosalicylic acid solution, and centrifuged at 3000 × *g* for 10 min, following which the inner layers were centrifuged at 10,000 × *g* for 10 min. The supernatant was passed through a 0.45 *μ*m filter, and amino acids and dipeptide (anserine and carnosine) were detected by the auto amino‐acid analyser (HITACHI L‐8900; Hitachi High‐Technologies Corporation, Tokyo, Japan). The fluid was separated using a #2622sc (PF) column (*ϕ*4.6 mm × 80 mm; Hitachi, Japan) by gradient elution with L‐8500 PF‐kit buffer according to the physiological fluids analysis method. Amino acids postlabeled with ninhydrin were detected by measuring the absorbance at 440 and 570 nm. Free amino acid and dipeptide content was examined at day 0.

### Sensory evaluations

Noninoculated raw meat samples were analysed for their freshness, texture, odor, spoilage/decay, and overall acceptability by nine subjects. Sensory qualities of the samples were evaluated using a five‐point scoring method. Sensory scores (freshness, odor, spoilage/decay, and overall acceptability) were 5: excellent, 4: very good, 3: good, 2: fair, and 1: poor. In texture analysis, each sample was pressed through plastic wrap by a finger. The texture scores were classified as 5 grade, 5: much hard, 4: stiff and hard, 3: slightly hard, 2: much soft and tender, and 1: very tender. All meat samples were served in Petri dishes. Sensory evaluation was examined at day 0 and up to 7 days of under 4°C storage condition. This sensory evaluations was conducted after receiving approval from the University of Shizuoka ethics committee (No. 25–42).

### Statistical analyses

All experiments were repeated at three times, on different days with different samples. Analyses were carried out replicate for each triplicate except for free amino acid and dipeptide analysis. Free amino acid and dipeptide values were the average of a result of two different samples.

Microbiological data were analyzed using 1‐factor ANOVA, and differences between individual group means were analyzed by using the Tukey‐Kramer test. The Steel‐Dwass test was used if the variance was unequal. Relative gene expression of SEA gene and some quality variables of the samples were analyzed with the *t* test using Microsoft Excel 2013. Results of all analyses showed as mean value ± standard deviation (SD). Differences in the means among treatments were determined by the least‐squares method (significance was defined at *P *<* *0.05).

## Results and Discussion

### Microbiological changes on boneless chicken breasts and beef liver treated with AlEW and StAEW

**Figure 1:**
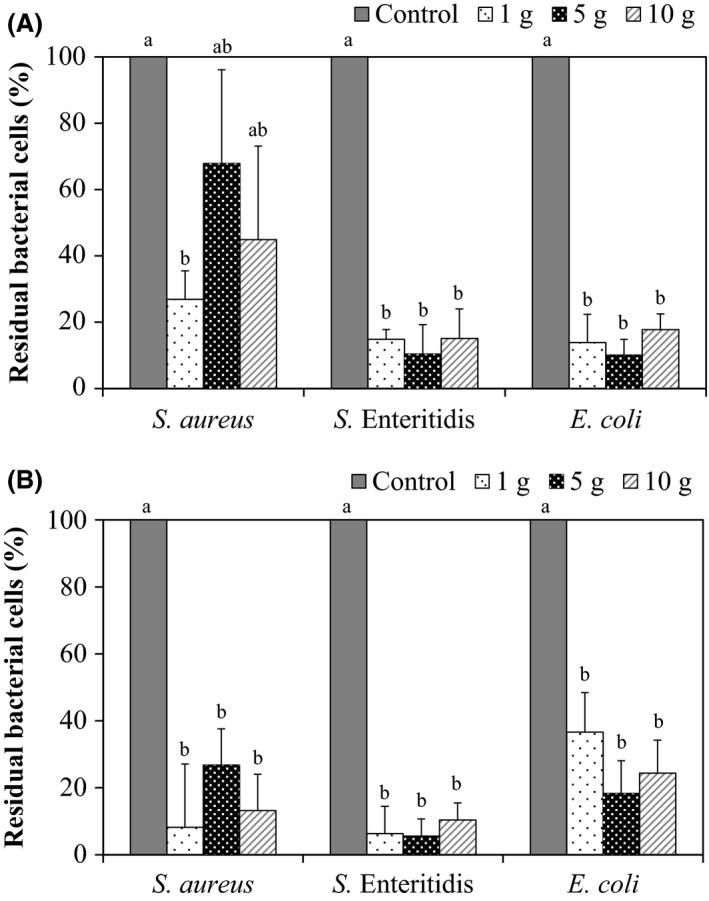
Effect of combined treatment with alkaline electrolysed water (AlEW) and strong acidic electrolysed water (StAEW) on *Salmonella* Enteritidis, *Escherichia coli*, and *Staphylococcus aureus* of fresh chicken breasts. StAEW treatments were performed at 4°C (A) and 25°C (B). Values represent the mean ± SD for three independent experiments. Means with the same letter are not significantly different from each other (Tukey‐Kramer test, *P *<* *0.05).

The sterilization effect of a combination treatment on bacteria (*S*. Enteritidis or *E*. *coli* or *S*. *aureus*) with AlEW and StAEW on chicken breasts was evaluated (Fig. 1). Initial counts of *S*. Enteritidis, *E*. *coli*, and *S*. *aureus* on 10 g of chicken breast after treatment with sterilized water (control) were 4.31, 4.24, and 4.17 log CFU/g (4°C) and 4.24, 4.26, and 3.70 log CFU/g (25°C) respectively. Combination treatment with AlEW and StAEW (4°C and 25°C) decreased the initial populations of *S. *Enteritidis, *E*. *coli*, and *S*. *aureus* on 10 g of chicken breast by 3.46, 3.43, and 3.74 log CFU/g (4°C) and 3.25, 3.63, and 3.92 log CFU/g (25°C) respectively. Initial counts of *S*. Enteritidis, *E*. *coli,* and *S*. *aureus* on 5 g of chicken breast after treatment with sterilized water (control) were 4.56, 4.67, and 4.31 log CFU/g (4°C) and 4.39, 4.59, and 3.76 log CFU/g (25°C) respectively. Combination treatment with AlEW and StAEW (4°C and 25°C) decreased the initial populations of *S*. Enteritidis, *E*. *coli,* and *S*. *aureus* on 5 g of chicken breast by 3.58, 3.68, and 3.21 log CFU/g (4°C) and 3.22, 3.11, and 3.20 log CFU/g (25°C) respectively. Initial counts of *S. *Enteritidi*s*,* E*. *coli,* and *S*. *aureus* on 1 g of chicken breast after treatment with sterilized water were 4.34, 4.45, and 4.36 log CFU/g (4°C) and 4.26, 4.34, and 4.17 log CFU/g (25°C) respectively. Combination treatment with AlEW and StAEW (4°C and 25°C) decreased the initial populations of *S*. Enteritidis, *E*. *coli,* and *S*. *aureus* on 1 g of chicken breast by 3.51, 3.63, and 3.97 log CFU/g (4°C) and 3.16, 3.12, and 3.14 log CFU/g (25°C) respectively. Complete elimination of bacteria on chicken breast using combination treatment with AlEW and StAEW is not possible in cases of a high degree of contamination.

**Figure 2:**
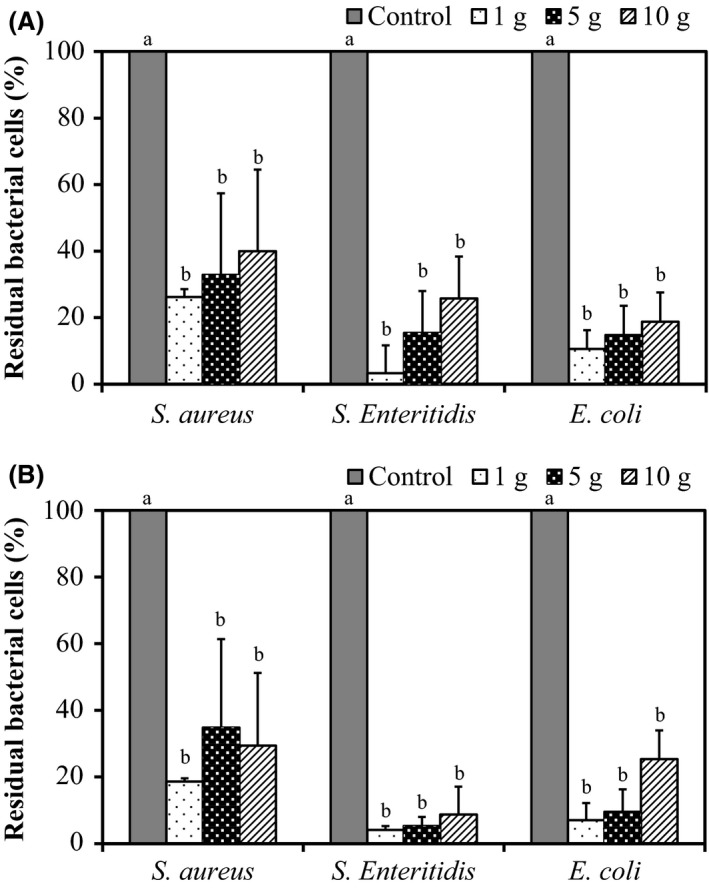
Effect of combined treatment with alkaline electrolysed water (AlEW) and strong acidic electrolysed water (StAEW) on *Salmonella* Enteritidis, *Escherichia coli*, and *Staphylococcus aureus* of fresh beef liver. StAEW treatments were performed at 4°C (A) and 25°C (B). Values represent the mean ± SD for three independent experiments. Means with the same letter are not significantly different from each other (Tukey‐Kramer test, *P *<* *0.05).

We then evaluated the sterilization effect of a combination treatment on bacteria with AlEW and StAEW on raw beef liver (Fig. 2). Initial counts of *S*. Enteritidis, *E*. *coli,* and *S*. *aureus* on 10 g of beef liver after treatment with sterilized water were 4.21, 4.29, and 4.14 log CFU/g (4°C) and 4.25, 4.28, and 4.40 log CFU/g (25°C) respectively. Combination treatment with AlEW and StAEW (4°C and 25°C) decreased the initial populations of *S*. Enteritidis, *E*. *coli*, and *S*. *aureus* on 10 g of beef liver by 3.54, 3.55, and 3.56 log CFU/g (4°C) and 3.22, 3.72, and 3.12 log CFU/g (25°C) respectively. Initial counts of *S*. Enteritidis, *E*. *coli*, and *S*. *aureus* on 5 g of beef liver after treatment with sterilized water were 4.53, 4.56, and 4.53 log CFU/g (4°C) and 4.49, 4.70, and 4.16 log CFU/g (25°C) respectively. Combination treatment with AlEW and StAEW (4°C and 25°C) decreased the initial populations of *S*. Enteritidis, *E*. *coli*, and *S*. *aureus* on 5 g of beef liver by 3.82, 3.83, and 3.17 log CFU/g (4°C) and 3.26, 3.66, and 3.54 log CFU/g (25°C) respectively. Initial counts of *S*. Enteritidis, *E*. *coli,* and *S*. *aureus* on 1 g of beef liver after treatment with sterilized water were 4.34, 4.36, and 4.46 log CFU/g (4°C) and 4.32, 4.30, and 4.18 log CFU/g (25°C) respectively. Combination treatment with AlEW and StAEW (4°C and 25°C) decreased the initial populations of *S*. Enteritidis, *E*. *coli*, and *S*. *aureus* on 1 g of beef liver by 3.12, 3.38, and 3.12 log CFU/g (4°C) and 3.13, 3.21, and 3.33 log CFU/g (25°C) respectively.

Combination treatment with AlEW and StAEW decreased the initial populations of bacteria on samples under both temperatures regardless of ACC content and weight. Combination treatment with AlEW and StAEW were more effective in decreasing initial populations of *S*. Enteritidis and *E*. *coli* than *S*. *aureus*. These results suggest that there is a difference in sensitivity between the gram‐positive bacteria and the gram‐negative bacteria on the AlEW and StAEW. The difference in weight of the samples (1, 5, and 10 g) did not influence the effectiveness of sterilization (Figs. 1, 2). These results suggest that sufficient sterilization effects were provided with 100 mL of each AlEW and StAEW for 10 g of samples. Rahman et al. (2012) reported that the reduction in *Listeria monocytogenes* and *Salmonella* Typhimurium on 10 g of fresh chicken breasts by 1.5 to 2.3 log CFU/g was achieved by the dipping treatment (22 ± 2°C for 10 min) with StAEW (Rahman et al. 2012). Fabrizio et al. (2002) demonstrated that spray‐washing (85 psi, 25°C, 15 sec) with electrolysed oxidizing water reduced the number of *S*. *typhimurium* to approximately 0.86 log CFU/g (Fabrizio et al. 2002). Similar results were obtained using AlEW and StAEW in the current study. In addition, because pH of the waste fluid becomes neutral (approximately pH 7.0) by continuously mixing AlEW (pH 11.5) with StAEW (pH 2.5), there is the advantage to the method pertaining to discarded waste liquid not needing any pH buffering treatment; therefore, the method is potentially environmentally friendly. Future research could focus on finding more effective washing methods using AlEW and StAEW to control food poisoning bacteria in meat.

### RT‐PCR

**Figure 3:**
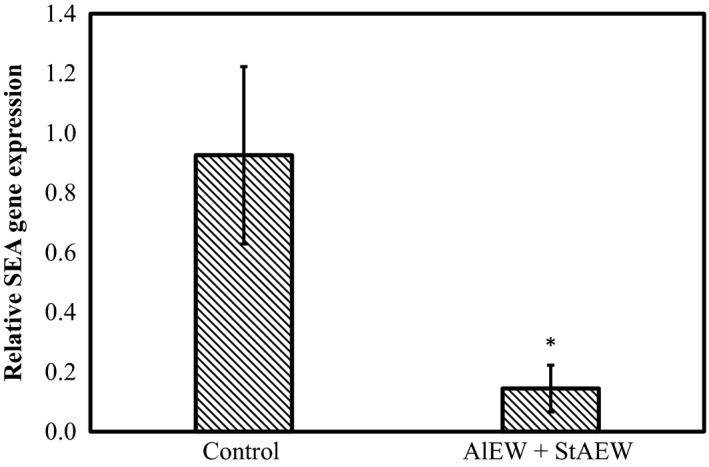
Relative gene expression of staphylococcal enterotoxin A (SEA) gene in *Staphylococcus aureus* C‐29 with or without combined treatment of alkaline electrolysed water (AlEW) and strong acidic electrolysed water (StAEW). Values represent the mean ± SD for three independent experiments. *Represents *P *<* *0.05 compared with sterilized water were used as a control.

Based on the finding that combination treatment with AlEW and StAEW decreased the initial populations of *S*. *aureus* on chicken breast and beef liver, we used a real‐time RT‐PCR assay to investigate the relative expression levels of *SEA* toxin‐encoding genes of *S*. *aureus* after combination treatment with AlEW and StAEW. SEA is produced early in conjunction with an exponential growth of *S*. *aureus* (Borst and Betley 1994). As shown in Figure 3, combination treatment with AlEW and StAEW significantly inhibited the transcription of SEA in *S*. *aureus* C‐29 during the stationary growth phase (*P *<* *0.05). The expression of the SEA gene was not detected in the control of the nontreated beef liver samples. Suzuki et al. (2002) reported that EW could eliminate SEA production in a brain heart infusion broth (Suzuki et al. 2002). In addition, EW treatment caused fragmentation of SEA. It was considered that EW treatment could denature SEA through an oxidative reaction caused by hydroxyl radicals and reactive chlorine (Vinci and Antonelli 2002). Although the complete sterilization of bacteria is very difficult, the inhibition in toxin expression of bacteria would be useful in preventing outbreaks of food‐borne disease. Our data demonstrate that combination treatment with AlEW and StAEW had an inhibitory effect on SEA production.

### Changes in pH

**Figure 4:**
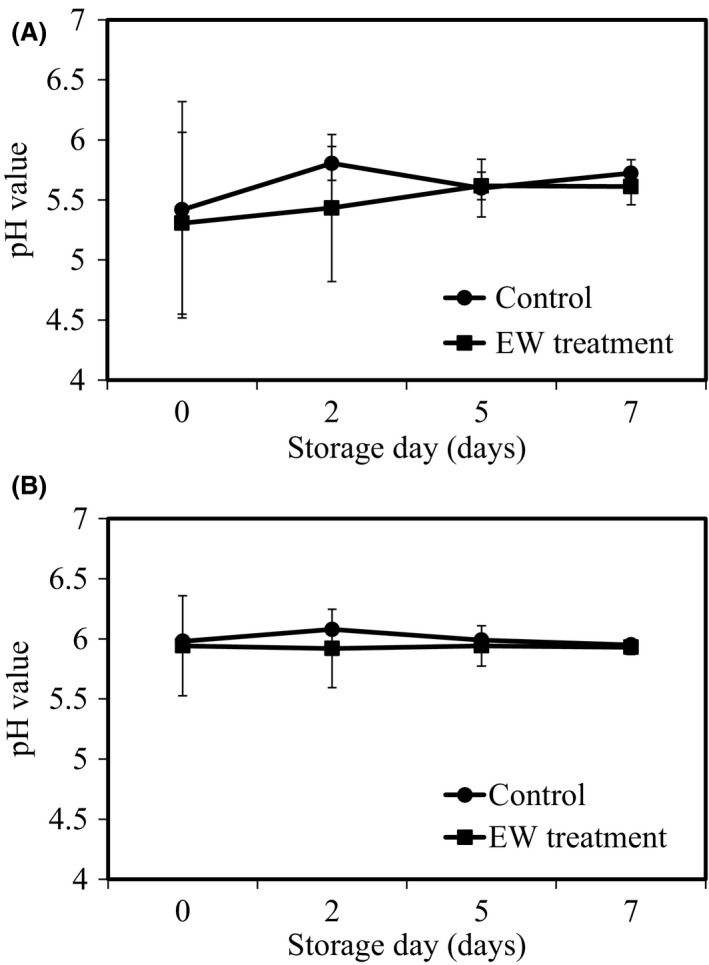
Changes in pH of samples with or without combined treatment of alkaline electrolysed water (AlEW) and strong acidic electrolysed water (StAEW). (A) Chicken breasts and (B) beef liver samples during storage at refrigeration temperature (4°C). Values represent the mean ± SD for six independent experiments.

The pH value of chicken meat samples with or without AlEW and StAEW treatment changed from 5.42 to 5.72 [Fig. 4(A)] and 5.31 to 5.61 [Fig. 4(B)] during storage at 4°C respectively. In the beef liver samples with or without AlEW and StAEW treatment, the pH value changed from 5.98 to 5.95 and 5.94 to 5.93 respectively. Rahman et al. (2012) reported that the mean pH of chicken meat samples was 5.9 and ranged from 5.8 to 6.7 during storage at 5°C for the control and StAEW treated samples respectively (Rahman et al. 2012). Similar results were obtained from in this study using combination treatment with AlEW and StAEW. In general, the pH value of meat increases with passage of storage time and following protein destructions and amine production (Lu and Wu 2012). It was suggested that increase of pH can be suppressed by AlEW treatment. These results suggest that increase of pH and following deterioration in quality of meat can be improved by combination treatment with AlEW and StAEW.

### Changes in lipid oxidation

**Figure 5:**
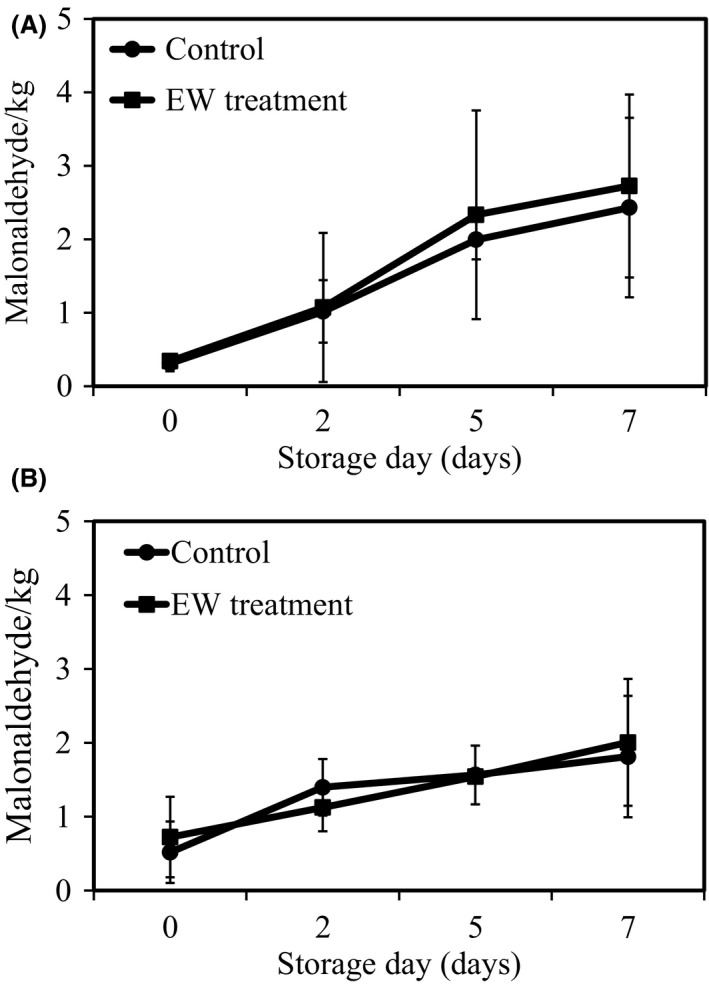
Changes in thiobarbituric acid‐reactive substances **(** TBARS) of samples with or without combined treatment of alkaline electrolysed water (AlEW) and strong acidic electrolysed water (StAEW). (A) Chicken breasts and (B) beef liver samples during storage at refrigeration temperature (4°C). Values represent the mean ± SD for six independent experiments.

The TBARS values as a measure of the degree of lipid oxidation of samples are shown in Figure 5. The TBARS values of chicken breast and beef liver samples with and without treatment by AlEW and StAEW changed from 0.31 to 2.43 [Fig. 5(A)] and 0.34 to 2.72 malonaldehyde (MA)/kg sample [Fig. 5(B)] during storage at 4°C respectively. The beef liver samples with or without treatment by AlEW and StAEW showed a change in the TBARS value from 0.51 to 1.81 and 0.72 to 2.0 MA/kg sample respectively. There was no significant difference in TBARS value between the control and treated samples. Rahman et al. (2012) reported that TBARS values of chicken breasts were increased by slightly acidic low concentration electrolysed water (10 ppm) and strong acidic electrolyzed water (50 ppm) treatments, varying between 0.8 and 3.2 MA/kg meat (Rahman et al. 2012). We have confirmed similar results by combination treatment with AlEW and StAEW in the present study. These results suggest that lipid oxidation of meat can be reduced by combination treatment with AlEW and StAEW.

### Changes in color

**Figure 6:**
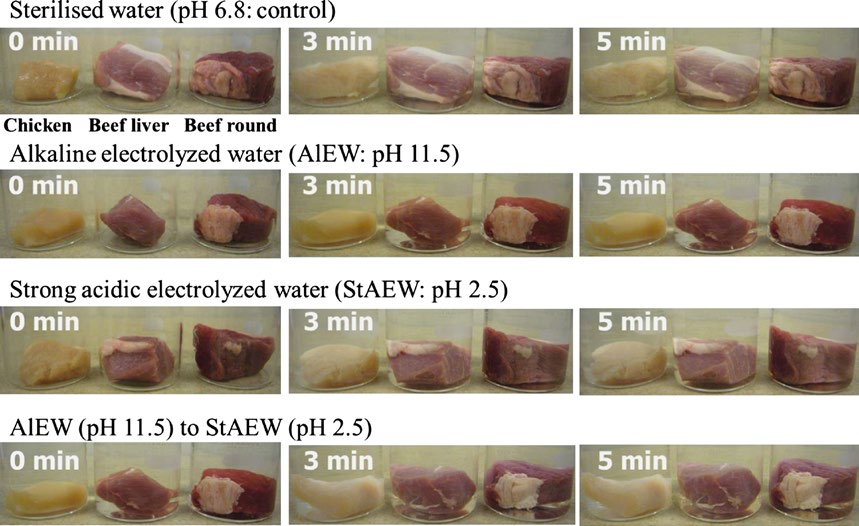
The effect of color of samples with combined treatment of alkaline electrolysed water (AlEW) and strong acidic electrolysed water (StAEW). Each inoculated sample was dipped in each 100 mL of AlEW and StAEW solutions for 0, 3, and 5 min.

The color of chicken breast and beef liver samples was confirmed by visual observation. The appearance of sample was not significantly changed among control (sterilized water) and treated samples (Fig. 6). In addition, the data are presented for each sample by change in color parameters for L*(brightness/darkness), a*(redness/greenness) and b* (yellowness/blueness) values (Table 1). Color variables were not significantly changed among untreated and treated samples. However, chicken breasts treated with AlEW and StAEW were brighter in color than untreated samples (*P *<* *0.05), with an average brightness of untreated chicken breasts being 51.50, whereas the brightness of treated chicken breasts was around 55.35. Lu and Wu (2012) reported that the lightness of chicken breasts treated by 200 ppm chlorine was 53.95 (Lu and Wu 2012). Yang and Froning (1992) demonstrated that washing solution containing 0.5% sodium bicarbonate, sodium phosphate buffer or 0.1 mol/L sodium chloride increased the lightness (L* values) of washed chicken meat (Yang and Froning 1992). In addition, Ellis et al. (2006) demonstrated that chlorine dioxide increased the lightness of washed chicken breast (Ellis et al. 2006). Because the color in the inside of the chicken breast was not changed by treatment with AlEW and StAEW (data not shown) in this study, the use of AlEW and StAEW appears practical.

**Table 1:** Changes in color parameters of chicken breasts, beef liver, and beef round with or without combination treatment of alkaline electrolysed water (AlEW) and strong acidic electrolysed water (StAEW)

Samples	0 day		2 days		5 days		7 days	
	Control	AlEW + StAEW	Control	AlEW + StAEW	Control	AlEW + StAEW	Control	AlEW + StAEW
Chicken breasts								
L*	50.19 ± 1.32	52.58 ± 1.84*	51.36 ± 1.23	54.29 ± 2.28	52.22 ± 0.71	60.29 ± 0.30*	52.22 ± 1.09	54.25 ± 1.29*
a*	5.38 ± 0.59	4.36 ± 0.61	2.6 ± 0.79	2.74 ± 0.61	3.35 ± 0.71	2.87 ± 0.81	4.65 ± 0.42	3.47 ± 1.11
b*	5.21 ± 0.38	3.88 ± 0.74	3.6 ± 0.51	4.41 ± 1.49	5.03 ± 0.61	5.85 ± 1.34	5.11 ± 0.48	5.17 ± 0.60
Beef liver								
L*	39.47 ± 0.49	39.65 ± 0.32	40.54 ± 0.79	40.6 ± 0.48	40.66 ± 1.26	41.54 ± 1.29	41.72 ± 1.24	42.19 ± 0.98
a*	10.85 ± 0.48	10.36 ± 0.37	11.13 ± 0.36	10.93 ± 0.45	10.0 ± 0.83	10.34 ± 0.84	10.61 ± 0.51	10.61 ± 0.51
b*	3.10 ± 0.34	2.81 ± 0.21	3.27 ± 0.32	2.88 ± 0.51	3.43 ± 0.60	3.2 ± 0.38	3.45 ± 0.38	3.29 ± 0.27
Beef round								
L*	42.63 ± 2.19	43.72 ± 2.07	44.1 ± 4.17	42.26 ± 1.23	44.18 ± 2.40	43.56 ± 1.34	45.88 ± 1.85	47.18 ± 2.51
a*	13.53 ± 1.36	13.01 ± 1.19	14.61 ± 1.08	13.89 ± 1.09	14.91 ± 1.75	13.36 ± 2.55	14.56 ± 1.41	11.67 ± 1.95*
b*	1.92 ± 0.37	1.63 ± 0.36	2.27 ± 1.09	1.50 ± 0.50	3.41 ± 1.60	3.27 ± 1.24	4.72 ± 0.82	5.87 ± 2.48

Color variables for L*(brightness/darkness), a* (redness/greenness) and b* (yellowness/blueness) values reported as mean ± standard deviation, *n* = 3 × 2.

*Represents *P *<* *0.05, compared to the control.

### Changes in free amino acid and dipeptide

**Figure 7:**
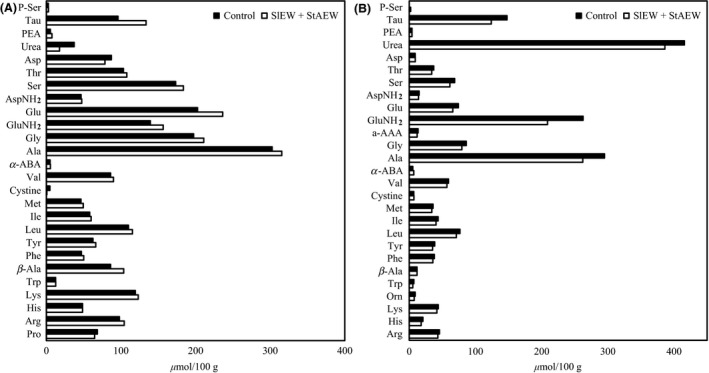
Changes in free amino acid concentration in samples with or without combined treatment of alkaline electrolysed water (AlEW) and strong acidic electrolysed water (StAEW). (A) Chicken breasts and (B) beef liver samples during storage at refrigeration temperature (4°C). Values are the average of two separate experiments.

The concentrations of free amino acids are affected by the quality of meat (Watanabe et al. 2004). Some of free amino acids contribute to the taste and nutrition of meat (Nishimura and Kato 1988). The amounts of amino acids in chicken breast and beef liver samples are showed in Figure 7. There was no significant difference in concentrations of free amino acids between the control and treated samples. In addition, contents of anserine and carnosine in untreated chicken breasts and beef liver samples were 1.85 and 1.37, respectively, and 0.18 and 1.46, respectively (nmol/100 g), whereas those of treated chicken breasts and beef liver samples were around 1.88 and 1.31, respectively, and 0.16 and 1.40 (nmol/100 g) respectively. These results suggest that free amino acid and dipeptide concentrations in meat samples are not changed by combination treatment with AlEW and StAEW.

### Sensory evaluations

Sensory evaluations (noninoculated) of chicken breast and beef liver samples are shown in Table 2. Sensory qualities were evaluated in the following items; freshness, texture, decay, and odor. There was no significant difference in sensory evaluations between the control and treated samples except for texture. After 7 days of storage, chicken meat and beef liver treated with AlEW and StAEW were chewier than nontreated samples. Salt contributes to water retention and firmness of meat products (Puolanne et al. 2001). Rahman et al. (2012) reported that the residual NaCl content of electrolysed water contributes to the freshness and color of meat samples; in addition, it contributes to maintaining meat odor (Rahman et al. 2012). Furthermore, these results suggest that the combination treatment with AlEW and StAEW is maintained good elastic of the meat samples. These results appear to demonstrate that the combination treatment with AlEW and StAEW could be used as a novel method for producing meat products characterized by a higher water retention and elasticity.

**Table 2:** Sensory evaluation of chicken breasts and beef liver with or without combination treatment of alkaline electrolysed water (AlEW) and strong acidic electrolysed water (StAEW) during storage at 4°C

Sensory attributes	Chicken breasts				Beef liver			
	0 day	2 days	5 days	7 days	0 day	2 days	5 days	7 days
Freshness								
Control	2.91 ± 0.4	2.9 ± 1.1	3.0 ± 0.7	2.9 ± 0.3	3.0 ± 0.3	3.0 ± 0.7	3.0 ± 1.0	3.2 ± 0.5
AlEW + StAEW	3.0 ± 0.9	2.9 ± 0.8	2.9 ± 0.7	2.6 ± 0.8	3.0 ± 0.0	3.0 ± 0.8	3.0 ± 0.9	2.7 ± 0.8
Texture								
Control	2.9 ± 0.9	2.8 ± 0.7	3.1 ± 0.9	3.2 ± 0.7	2.9 ± 0.5	2.9 ± 0.7	2.9 ± 0.7	3.0 ± 0.5
AlEW + StAEW	3.5 ± 1.1	3.4 ± 1.0*	3.6 ± 0.8*	3.5 ± 1.0	3.5 ± 0.8*	3.5 ± 0.8*	3.8 ± 0.8*	3.5 ± 0.7*
Decay								
Control	3.0 ± 0.0	3.1 ± 0.8	3.0 ± 0.5	2.9 ± 0.4	3.0 ± 0.0	3.1 ± 0.8	3.1 ± 0.9	3.3 ± 0.7
AlEW + StAEW	3.3 ± 0.6	3.1 ± 0.6	3.0 ± 0.6	2.8 ± 0.8*	3.1 ± 0.2	3.3 ± 0.8	2.6 ± 0.7	2.9 ± 0.8
Odor								
Control	3.0 ± 0.5	3.0 ± 0.5	2.9 ± 0.7	2.9 ± 0.4	3.0 ± 0.8	2.9 ± 0.4	3.0 ± 0.8	3.1 ± 0.3
AlEW + StAEW	2.9 ± 0.6	3.0 ± 0.4	2.8 ± 0.4	2.7 ± 0.6	2.9 ± 0.8	2.8 ± 0.6	2.8 ± 0.6	2.9 ± 0.5
Overall								
Control	3.0 ± 0.5	3.0 ± 0.8	3.0 ± 0.6	3.0 ± 0.5	3.0 ± 0.3	3.0 ± 0.7	3.0 ± 0.9	3.2 ± 0.5
AlEW + StAEW	3.2 ± 0.9	3.2 ± 0.8	2.9 ± 0.7*	3.0 ± 0.7	3.0 ± 0.6	3.2 ± 0.9	2.9 ± 0.6	2.9 ± 0.9

*Represents: *P *<* *0.05, compared to the control. Sensory scores were based on a five‐point descriptive scale, where 1 = poor, 2 = fair, 3 = good, 4 = very good and 5 = excellent.

## Conclusion

The combination treatment with AlEW and StAEW on chicken breasts and beef liver significantly reduced bacteria, with a reduction of about 1 log CFU/g achieved. In addition, this combination treatment significantly inhibited the transcription of bacterial toxin in chicken breasts and beef liver. There were no difference between treated with AlEW and StAEW samples and untreated samples in some meat quality variables such as pH, lipid oxidation, color, and amino‐acid content. These results show that combination treatments with AlEW and StAEW is the useful sterilization method to extend the microbiological shelf life of chicken meat and beef liver without deteriorating its quality. Currently in the slaughter treatment of Japan, a dressed carcass is washed with tap water. Therefore, by using AlEW and StAEW in place of the tap water, it is considered that microbiological safety of meat can become increasingly. In the future, the combination treatment with AlEW and StAEW is expected to improve food safety in various food manufacturing domains.

## Conflict of Interest

The authors have declared no conflicts of interest.
